# Long Non-coding RNA SNHG17 Upregulates RFX1 by Sponging miR-3180-3p and Promotes Cellular Function in Hepatocellular Carcinoma

**DOI:** 10.3389/fgene.2020.607636

**Published:** 2021-01-15

**Authors:** Tao Ma, Xujun Zhou, Hailiang Wei, Shuguang Yan, Yi Hui, Yonggang Liu, Hui Guo, Qian Li, Jingtao Li, Zhanjie Chang, Xiao-Xin Mu

**Affiliations:** ^1^Department of Clinical Laboratory, Hospital of Chengdu University of Traditional Chinese Medicine, Chengdu, China; ^2^Department of Gastroenterology, Wuhan Eighth Hospital, Wuhan, China; ^3^Department of General Surgery, The Hospital Affiliated to Shaanxi University of Chinese Medicine, Xianyang, China; ^4^College of Basic Medicine, The Shaanxi University of Chinese Medicine, Xianyang, China; ^5^Department of Liver Diseases, The Hospital Affiliated to Shaanxi University of Chinese Medicine, Xianyang, China; ^6^Medical Experiment Center, The Shaanxi University of Chinese Medicine, Xianyang, China; ^7^Key Laboratory of Liver Transplantation, Chinese Academy of Medical Sciences, Nanjing, China; ^8^National Health Council (NHC) Key Laboratory of Living Donor Liver Transplantation, Hepatobiliary Center, The First Affiliated Hospital of Nanjing Medical University, Nanjing, China

**Keywords:** lncRNA, SNHG17, miR-3180-3p, RFX1, hepatocellular carcinoma

## Abstract

**Background:**

Hepatocellular carcinoma (HCC) is one of the most common types of cancer that is associated with poor quality of life in patients and a global health burden. The mechanisms involved in the development and progression of HCC remain poorly understood.

**Methods:**

Hepatocellular carcinoma human samples and cell lines were subjected to qRT-PCR for expression assessment. CCK-8 assay, Transwell migration and invasion assay, were applied for cell function detection. Animal experiment was used to measure the function of SNHG17 on cell growth *in vivo*. Western blot was conducted to evaluate the level of EMT in cells. RIP, RNA pull-down and luciferase reporter assays were performed to assess the correlation between SNHG17, miR-3180-3p and RFX1.

**Results:**

Our study demonstrated that SNHG17 was upregulated in HCC human samples and involved cell proliferation, migration, invasion progress. SNHG17 promoted HCC cell growth and metastasis *in vivo*. Furthermore, we investigated the downstream factor of SNHG17, SNHG17 acted as a molecular sponge for miR-3180-3p, and SNHG17 regulated RFX1 expression via miR-3180-3p. SNHG17 promotes tumor-like behavior in HCC cells via miR-3180-3p/RFX1.

**Conclusion:**

We determined RFX1 as the target of miR-3810-3p; SNHG17 enhanced the progression of HCC via the miR-3180-3p/RFX1 axis. Taken together, our findings may provide insight into the molecular mechanism involved in the progression of HCC and develop SNHG17 as a novel therapeutic target against HCC.

## Introduction

Hepatocellular carcinoma (HCC) is the most common subtype of liver cancer (Mcglynn et al., 2015) and is the third cause for cancer-related mortality worldwide (Bray et al., 2018). Reportedly, half of the total global incidences of HCC can be found in China (Lafaro et al., 2015). The widespread risk factors for HCC include hepatitis C virus infection, alcohol consumption, obesity, and metabolic disorders and result in a high rate of morbidity. Owing to the rapidly progressing and metastasizing nature of HCC, majority of patients are diagnosed at later stages. Moreover, high rates of postsurgical recurrence leads to poor outcomes in patients with HCC (Margini and Dufour, 2016). Moreover, the limited treatment available for HCC has resulted in a poor quality of life for patients and a global health burden. Thus, it is imperative to identify novel therapeutic targets for HCC. However, the underlying mechanisms involved in the development and progression of HCC remain to be fully understood.

Long non-coding RNAs (lncRNAs) are > 200 nucleotide-long transcripts that do not encode proteins (Mercer et al., 2009; Liz and Esteller, 2016). LncRNAs have different expression profiles and biological functions in various diseases, especially cancers (Wang et al., 2010; Jarroux et al., 2017; Mathy and Chen, 2017), suggesting the crucial role of lncRNAs in cancer progression. Moreover, lncRNAs induce differential lncRNA-miRNA-mRNA network signatures (Zhang et al., 2016; Fan et al., 2018; Zhang Y. et al., 2018). LncRNAs have recently been implicated in the progression of HCC. Liu et al. showed that lncRNA NEAT-1 promotes the proliferation of HCC cells (Liu et al., 2018). Huang et al. demonstrated that lncRNA PTTG3P regulates HCC progression (Huang J.L. et al., 2018). Yang et al. reported that the lncRNA HOTAIR stimulates HCC progression (Yang et al., 2019). Thus, lncRNAs may be pivotal in the development and progression of HCC.

The lncRNA small nucleolar RNA host gene 17 (SNHG17), located on chromosome 20q11.23, was detected in patients with colorectal cancer. SNHG17 binds to EZH2 and suppresses p57 to stimulate the development of colorectal cancer (Ma et al., 2017). SNHG17 is also involved in the progression of gastric carcinoma (Chen et al., 2019), non-small cell lung cancer (Xu et al., 2019), type 2 diabetes mellitus (Mohamadi et al., 2019), and melanoma (Gao et al., 2019). However, the role of SNHG17 in HCC progression remains unclear.

Based on the literature, we hypothesized that SNHG17 plays a role in HCC progression and exerts its functions via a lncRNA-microRNA-mRNA regulatory network. We performed *in vitro* and *in vivo* experiments to show that SNHG17 was dysregulated in HCC tissues. Bioinformatic analyses, RNA immunoprecipitation (RIP) assays, RNA pull-down assays, and luciferase reporter assays showed that SNHG17 promoted tumor-like behavior in HCC cells via the miRNA-mRNA pathway. Taken together, our findings might help develop SNHG17 as a novel therapeutic target for HCC.

## Materials and Methods

### Clinical Samples

HCC tumor tissues and paired normal tissues were harvested from patients who were diagnosed with HCC based on pathological evaluation; patients underwent curative surgery at The Hospital Affiliated to Shaanxi University of Chinese Medicine between 2015 and 2018 (Table 1). None of the patients were administered with therapy targeting HCC before surgery. All the specimens were stored at −80°C until further use. This study was approved by the Ethical Review Committees at The Hospital Affiliated to Shanxi University of Chinese Medicine, and all the patients provided written informed consent. Tumor size and Edmonson-Steiner grade were confirmed by histopathology.

**TABLE 1:** Characteristics of hepatocellular cancer patients.

**Characteristics**		**Cases**
Age	<60	10
	≥60	13
Gender	Male	18
	Female	5
Size(cm)	<5	13
	≥5	10
Edmonson-Steiner grade	I–II	9
	III–IV	14
Vascular invasion	Yes	10
	No	13
AFP (ng/ml)	<200	9
	≥200	14
Histologic grade	Low	3
	Middle	12
	High	8

### Cell Culture and Transfection

All HCC cell lines and human non-cancerous hepatic cell line L02 were obtained from the American Type Culture Collection (Manassas, United States) and cultured in minimum essential medium (Logan, United States) with 10% fetal bovine serum (Logan, United States) at 37°C in 5% CO_2_ and 95% air environment. SNHG17 shRNA and siRNA, miR-3180-3p inhibitor and mimic, and control and RFX1 siRNAs were synthesized by GeneChem (Shanghai, China) and transfected into cells using Lipofectamine 2000 (Invitrogen, MA) following the prescribed protocol.

### Quantitative Reverse Transcription-Polymerase Chain Reaction (qRT-PCR)

Total RNA was extracted from cells using TRIzol (Invitrogen, Carlsbad, CA, United States) and reverse transcribed using the RevertAid First Strand cDNA Synthesis kit (Thermo Fisher Scientific, United States). Real-time PCR Master Mix (SYBR Green; TOYOBO, Japan) was used to perform qRT-PCR. GAPDH served as the internal control. Relative expression (fold change) of the target genes were calculated by the 2^–ΔΔ*Ct*^ method. Table 2 lists the primers used for qRT-PCR.

**TABLE 2:** Primers applied in study.

**Primers**	**Forward (5′∼3′)**	**Reverse (5′∼3′)**
LncSNHG17	TTTTCCCACGCTGTCTGTCA	CAGTTTCCCCCGATGGTGAG
miR-3180-3p	CGTCTAGAAAAAATCTAT GTTGGTTCGATAC	CGGCGGCCGCTAAATTCAGGAC GCGATCGAAG
RFX1	GATCCAAGGCGGCTACAT	CAGCCGTCTCATAGTTGTCC
GAPDH	ATGGGGAAGGTGAAGGTCG	GGGGTCATTGATGGCAACAATA

### Cell Counting Kit 8 (CCK-8)

Cell proliferation was analyzed using CCK-8 (Dojindo, Japan) as per the kit instructions. Collectively, about 1 × 10^3^ cells were seeded and cultured in a 96-well plates for 24, 48, 72, 96, and 120 h. Subsequently, cells were treated with 10 μl of CCK-8 assay solution for 2 h, and the proliferative capacity of treated cells were measured at 450 nm by an enzyme immunoassay analyzer (Thermo Fisher Scientific, United States).

### Transwell Migration Assay

Cell migration was measured using 24-well culture plates with 8 mm pore-containing membrane inserts. Serum-free cell-containing medium (Logan, United States) was added to the upper chamber and the lower chamber contained Dulbecco’s modified Eagle medium supplemented with 15% fetal bovine serum (Gibco, Grand Island, NY, United States). This was incubated at 37°C for 3 days. Cells in the lower chamber (below the membrane) were stained with 0.4% trypan blue (Invitrogen) and counted under a light microscope (×20 magnification). Each experiment was performed at least in triplicates.

### Western Blotting

Protein extraction reagent (Beyotime) was used to isolate tumor proteins and RIPA lysis buffer (Invitrogen) was used for cellular proteins. The proteins were separated by sodium dodecyl sulfate-polyacrylamide gel electrophoresis followed by transferring onto a polyvinylidene fluoride membrane (Invitrogen). The membrane was blocked at room temperature for 2 h with shaking following which it was incubated overnight with the primary antibody at 4°C followed by the secondary antibody (1:2,000 dilution) for 2 h. The bands corresponding to the proteins were detected and imaged using (Bio-Rad, United States). The antibodies we used in the study as following: E-cadherin (CST, 14472s), Vimentin (CST, 5741s), RFX1 (Abcam, ab244484), GAPDH (CST, 5174s).

### Tumor Xenograft

Five-weeks-old male/female nude BALB/7 mice (*n* = 30) were procured from Beijing Vital River Laboratory Animal Technology (Beijing, China). The mice were housed at 25°C with free access to food and water. All animal procedures were approved by The Hospital Affiliated to Shaanxi University of Chinese Medicine. The mice were randomly divided to the experimental and control groups: the experimental group was injected with treated Huh7 and HepG2 cells and control mice were injected with control cells via tail vein following which the mice were sacrificed. All the subcutaneous tumors and lungs were excised to measure tumor growth, size, weight, and metastasis.

### Immunohistochemistry

Sections (5 μm) were treated with formalin for immunohistochemical analysis. Tissue sections were incubated overnight with antibodies against E-cadherin (CST, 14472s) and vimentin (CST, 5741s) at 4°C. Scale bar represents 50 μm. The protocols for immunohistochemistry were performed as described previously (Zhang X.P. et al., 2018).

### Hematoxylin and Eosin Staining

The paraffin sections were pretreated by dewaxing according to conventional methods followed by hydrating and soaking with xylene as per the instructions of the Hematoxylin-Eosin staining kit (GeneChem, China).

### RNA-Binding Protein Immunoprecipitation Assay

The Magna RIP RNA-Binding Protein Immunoprecipitation Kit (Millipore, Massachusetts, United States) was used to perform RIP. Cells were lysed using the RIP lysis buffer (Invitrogen). Magnetic beads (Millipore) conjugated with AGO2 or control IgG antibody were incubated with the cell lysates along with Proteinase K (Millipore). The immunoprecipitated RNA was used for PCR analysis.

### Dual-Luciferase Reporter Assay

pmirGLO dual-luciferase reporter plasmids containing the wild-type (wt) or mutant (mt) forms of the 3’ untranslated region of SNHG17 or RFX1 were synthesized by GeneChem (Shanghai, China). These constructs and control plasmids were transfected into 293T cells. Luciferase activity was measured using the Dual-Luciferase Reporter Assay System (Promega) according to the kit instructions.

### RNA Pull-Down Assay

Biotinylated SNHG17 or miR-3180-3p probes and its controls were synthesized by GeneChem (Shanghai, China) and transfected into 293T cells for 48 h. Cell lysates were incubated with Dynabeads M-280 Streptavidin (Invitrogen, United States) at 4°C for 3 h. Ice-cold lysis buffer was used to wash the beads three times following the kit instructions. Subsequently, PCR was used to analyze the bound RNAs.

### Statistical Analysis

GraphPad Prism 6.0 was used for data analysis. Data are represented as mean ± standard deviation. Student’s *t*-test to compare data from different groups. The differences represented by ^∗^, ^∗∗^, and ^∗∗∗^ had *p*-values of 0.05, 0.01, and 0.001, respectively.

## Results

### SNHG17 Levels in HCC Tissues

**FIGURE 1:**
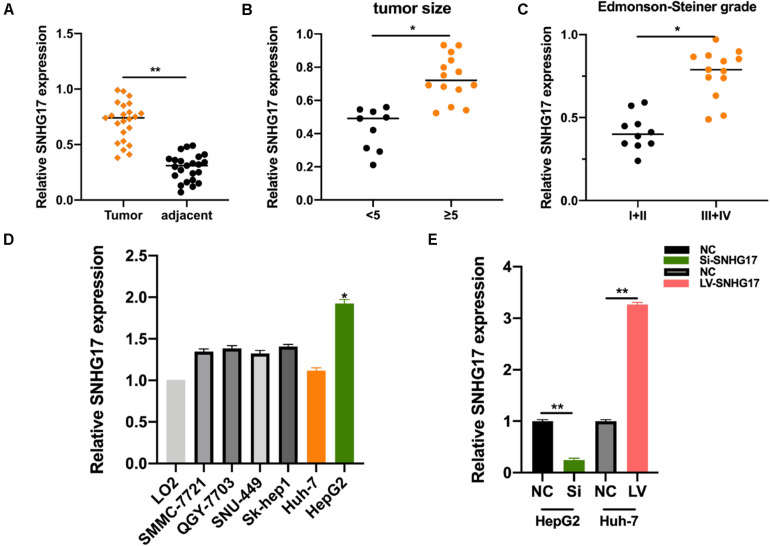
SNHG17 expression in HCC tissues. **(A)** Polymerase chain reaction (PCR) analysis for the expression of SNHG17 in HCC tumor and matched normal tissues. **(B)** SNHG17 levels in HCC tumors with varying size. **(C)** SNHG17 levels in HCC tumors with varying Edmonson-Steiner grades. **(D)** SNHG17 expression in different HCC cell lines. **(E)** Relative expression of SNHG17 in HepG2 cells transfected with si-SNHG17 and its controls and Huh7 cells transfected with LV-SNHG17 and its control plasmids. Error bars represent mean ± SD from at least three experiments. *p < 0.05, **p < 0.01.

We measured SNHG17 levels in HCC tumor and matched normal tissues. SNHG17 was significantly overexpressed in tumor tissues compared to that in the matched healthy tissues (Figure 1A). We then determined the expression of SNHG17 in HCC tumors of different sizes and Edmonson-Steiner grades. We observed high expression of SNHG17 with increasing tumor size and Edmonson-Steiner grade (Figures 1B,C). Based on these results, we speculated that SNHG17 is involved in the progression of HCC. Thus, we determined the expression of SNHG17 in HCC cell lines: SNHG17 was overexpressed in HepG2 cells and downregulated in Huh7 cells (Figure 1D). Thus, for our subsequent experiments, we depleted HepG2 cells of SNHG17 and overexpressed SNHG17 in Huh7 cells (Figure 1E).

### SNHG17 Promotes HCC Cellular Function

**FIGURE 2:**
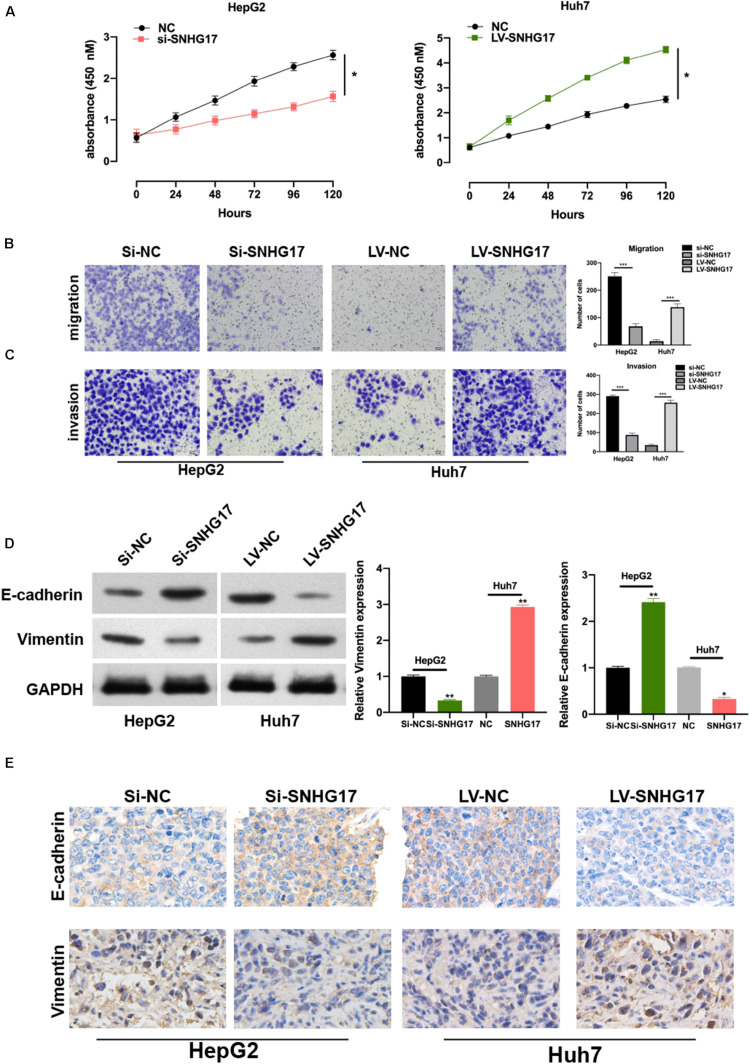
SNHG17 promotes HCC cell functions. **(A)** Proliferation of si-SNHG17-transfected HepG2 cells and LV-SNHG17-transfected Huh7 cells using the cell counting kit-8 (CCK-8). **(B)** Transwell migration assay was used to evaluate the migration of si-SNHG17-transfected HepG2 cells and LV-SNHG17-transfected Huh7 cells. **(C)** Potential for invasion by si-SNHG17-transfected HepG2 cells and LV-SNHG17-transfected Huh7 cells as assayed by the Transwell migration assay. **(D)** PCR and Western blotting for the expression of E-cadherin and vimentin in treated cells. **(E)** Representative images for the immunohistochemical analysis of the expression of E-cadherin and vimentin in human HCC tumor tissues, Scale bar: 20 μm. Error bars represent the mean ± SD from at least three experiments. *p < 0.05, **p < 0.01, and ***p < 0.001.

To investigate the role of SNHG17 in the progression of HCC, we generated SNHG17 overexpressing and depleted cells and rat models. SNHG17 overexpression promoted cell proliferation (Figure 2A) and enhanced cell migration and invasion (Figures 2B,C). Epithelial–mesenchymal transition (EMT) is crucial in metastasis (Lo and Zhang, 2018; Pastushenko and Blanpain, 2019). Overexpression of SNHG17 stimulated EMT in HCC (Figure 2D). Immunohistochemistry revealed increased staining for E-cadherin and vimentin in rat tumor tissues as compared to that in the matched normal tissues, suggesting that upregulation of SNHG17 stimulated EMT in HCC (Figure 2E).

**FIGURE 3:**
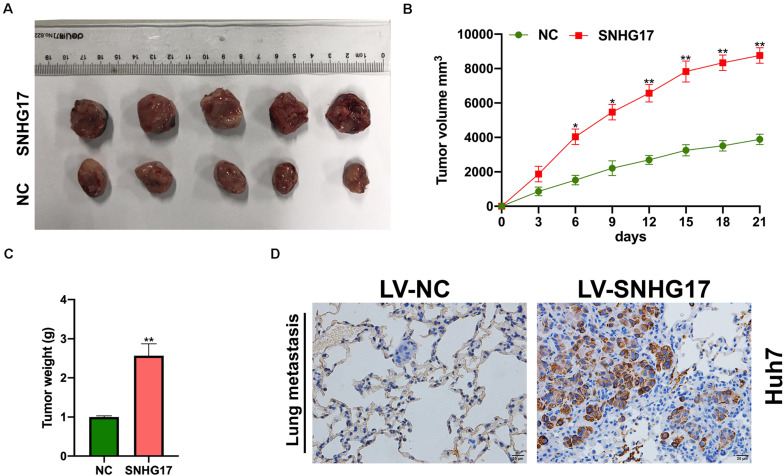
Effect of SNHG17 on tumor volume and weight. **(A)** Representative images of the subcutaneous tumors isolated from mice. Scale bar: 1 cm. **(B)** Subcutaneous tumor volume was measured every 3 days after inoculation. **(C)** Subcutaneous tumor weight was measured on day 21 after inoculation. **(D)** Hematoxylin and eosin staining of lung metastases, Scale bar: 20 μm. Error bars represent mean ± SD from at least three experiments. *p < 0.05, **p < 0.01.

Next, we isolated the subcutaneous tumors from mice and measured the volume and weight. SNHG17 overexpression resulted in increased tumor growth *in vivo* (Figures 3A–C). Hematoxylin and eosin staining of lung tissues from mice with HCC revealed overexpression of SNHG17 promoted tumor metastasis (Figure 3D).

### SNHG17 Sponges miR-3180-3p

**FIGURE 4:**
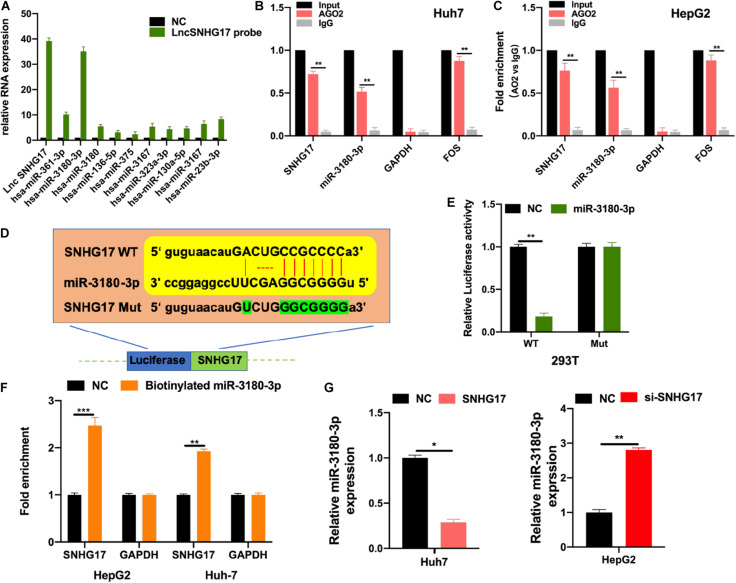
SNHG17 sponges miR-3180-3p. Bioinformatic analysis were conducted to predicted putative targets for SNHG17 (ENCORI database: http://starbase.sysu.edu.cn/index.php; and CLIP Data with high stringency ≥ 3). **(A)** HepG2 cell lysates were used for biotinylated RNA pull-down. Purified RNA was analyzed by PCR using specific miRNA primers. **(B,C)** RIP using antibodies against AGO2, IgG, GAPDH (negative control), and FOS (positive control). **(D)** Binding site for SNHG17 on miR-3180-3p as predicted by bioinformatics (ENCORI, LncBase predicted V2). **(E)** Luciferase activity of transfected 293T cells. **(F)** Biotinylated miR–3180–3p was transfected into cells with SNHG17 overexpression. SNHG17 expression was detected by quantitative real–time PCR, after streptavidin capture. **(G)** Relative expression of miR-3180-3p in si-SNHG17-transfected HepG2 cells and LV-SNHG17-transfected Huh7 cells. Error bars represent mean ± SD from at least three experiments. *p < 0.05, **p < 0.01, ***p < 0.001.

LncRNAs bind to and sponge miRNAs to regulate their function. Thus, we used bioinformatic analysis to identify the top 10 miRNAs for further analysis. We performed RNA pull-down to show the extensive enrichment of miR-3180-3p in SNHG17 immunoprecipitated samples (Figure 4A). Next, we performed RIP using AGO2 as the bait in Huh7 and HepG2 cells. As shown in Figures 4B,C, SNHG17 and miR-3180-3p (not GAPDH) were enriched in the AGO2 immunoprecipitated samples. Subsequently, we analyzed the binding sites between SNHG17 and miR-3180-3p (Figure 4D). Luciferase reporter assays showed that overexpressing miR-3180 decreased luciferase activity in cells with wt SNHG17, but increased luciferase activity in cells containing the mt form of SNHG17 (Figure 4E). RNA pull-down assays demonstrated the enrichment of SNHG17 in cells transfected with biotinylated miR-3180-3p mimics (Figure 4F), suggesting that SNHG17 sponges miR-3180-3p. Furthermore, overexpression of SNHG17 inhibited miR-3180-3p expression in HCC cells (Figure 4G).

### miR-3180-3p Reverses the Oncogenic Function of SNHG17

**FIGURE 5:**
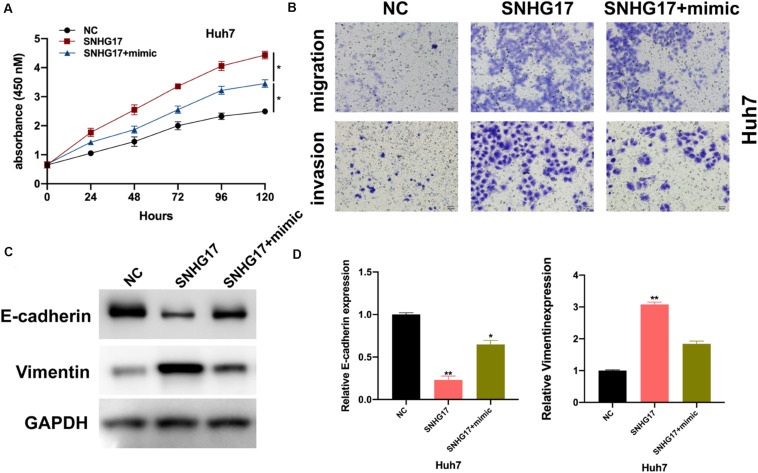
miR-3180-3p reverses the oncogenic effect of SNHG17. Determination of the target of miR-3180-3p was performed using miRmap: https://mirmap.ezlab.org/. **(A)** Proliferation of Huh7 cells transfected with SNHG17 and SNHG17+miR-3180-3p mimic using CCK-8. **(B)** Transwell migration assay for the migration and invasion of Huh7 cells transfected with SNHG17 and SNHG17+miR-3180-3p mimic. **(C,D)** Western blotting and PCR analysis for the expression of EMT biomarkers E-cadherin and vimentin in treated cells. Error bars represent mean ± SD from at least three experiments. *p < 0.05, **p < 0.01.

We wanted to determine the role of miR-3180-3p in the development and progression of HCC. Firstly, we assessed the effects of miR-3180-3p on HCC cellular progression. As shown in Supplementary Figure S1, it was found that miR-3180-3p inhibited HCC cell proliferation, migration, and invasion. Subsequently, we used CCK-8 and Transwell migration assays to analyze proliferation, migration, and invasion of Huh7 cells transfected with SNHG17 and SNHG17+miR-3180-3p mimics. miR-3180-3p mimics reversed the enhanced proliferation, migration, and invasion phenotype of HCC cells observed with SNHG17 overexpression (Figures 5A,B). SNHG17 and miR-3180-3p mimic co-transfected Huh7 cells showed reduced EMT as compared to that observed in SNHG17-transfected Huh7 cells (Figures 5C,D).

### SNHG17 Promotes Tumor-Like Behavior in HCC Cells via miR-3180-3p/RFX1

**FIGURE 6:**
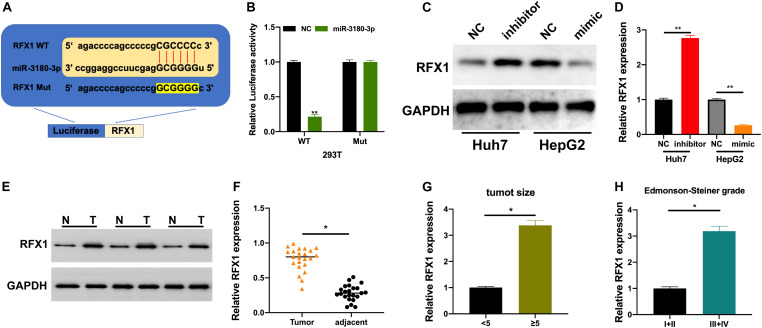
RFX1 overexpression promotes the progression of HCC. **(A)** Binding site for miR-3180-3p on RFX1. **(B)** Luciferase activity for the effect of miR-3180-3p on RFX1. **(C,D)** Western blotting and PCR for the relative expression of RFX1 in Huh7 and HepG2 cells transfected with miR-3180-30 control, inhibitor, and mimic. **(E)** Western blotting for the expression of RFX1 in HCC tumor and matched normal tissues. **(F)** RFX1 expression in HCC tumor and matched normal tissues. **(G)** RFX1 expression in HCC tumor tissues of varying size. **(H)** RFX1 expression in HCC tumor tissues of varying Edmonson-Steiner grade. Error bars represent mean ± SD from at least three experiments. *p < 0.05, **p < 0.01.

We used bioinformatic tools to further understand the underlying molecular mechanisms employed by miR-3180-3p in the progression of HCC progression. RFX1 is involved in the progression of various diseases (Wang et al., 2018; Du et al., 2019) and a potential target of miR-3180-3p. Thus, we generated plasmids for the wt and mt versions of RFX1 and miR-3180-3p mimic (Figure 6A). Luciferase assays showed a drastic reduction in luciferase activity in 293T cells containing miR-3180-3p and wt RFX1, but not in cells containing mt RFX1 (Figure 6B). Next, we measured RFX1 levels in Huh7 and HepG2 cells transfected with miR-3180-30 inhibitor, mimic, and controls. miR-3180-3p inhibited RFX1 expression and downregulation of miR-3180-3p promoted RFX1 expression in HCC cells (Figures 6C,D). Similarly, RFX1 was upregulated in HCC tumor tissues as compared to that in the normal tissues (Figures 6E,F); these high levels of RFX1 correlated with increasing tumor size and grade (Figures 6G,H).

**FIGURE 7:**
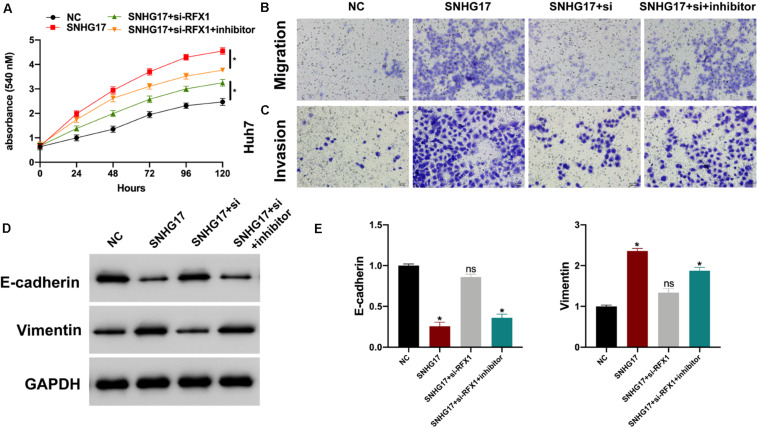
**(A)** Proliferation of Huh7 cells transfected with SNHG17, SNHG17+si-RFX1, and SNHG17+si-RFX1+inhibitor using CCK-8. **(B)** Transwell migration assay for the migration and invasion of Huh7 cells transfected with SNHG17, SNHG17+si-RFX1, SNHG17+si-RFX1+inhibitor. **(C,D)** Western blotting and PCR analyses for the expression of EMT biomarkers E-cadherin and vimentin in treated cells. **(E)** The SNHG17-miR-3180-3p-RFX1 axis in the progression of HCC. Error bars represent mean ± SD from at least three experiments. *p < 0.05.

Finally, we investigated the role of the SNHG17-miR-3180-3p-RFX1 axis in the progression of HCC using control, SNHG17, SNHG17+si-RFX1, and SNHG17+si-RFX1+miR-3180-3p inhibitor transfected Huh7 cells. SNHG17 promoted HCC cell proliferation (Figure 7A), migration (Figure 7B), invasion (Figure 7C), and EMT (Figures 7D,E) by sponging miR-3180-3p and upregulating RFX1.

## Discussion

LncRNAs have been implicated in the progression of various diseases, including HCC (Mehra and Chauhan, 2017; Lim et al., 2019). The role and molecular mechanisms employed by the lncRNA SNHG17 have been studied in detail; however, excluding its function in HCC. In this study, we hypothesized that SNHG17 is involved in the progression of HCC. SNHG17 was upregulated in HCC tumor tissues as compared to that in parameter-matched normal tissues. Moreover, high expression of SNHG17 correlated with larger tumor size and higher Edmonson-Steiner grades, suggesting that SNHG17 participates in the progression of HCC.

SNHG17 promotes cell proliferation in colorectal cancer (Ma et al., 2017), regulates cell invasion and migration in breast cancer (Du et al., 2020), and affects cell cycle in gastric cancer (Zhang et al., 2019). Thus, SNHG17 may be involved in the lifecycle of HCC cells. Here, we generated SNHG17 overexpressing and depleted cell and mouse models. Overexpression of SNHG17 promoted cell proliferation, invasion, and migration *in vitro*. Similarly, overexpressing SNHG17 promoted tumor growth and metastasis *in vivo*. Moreover, upregulating SNHG17 promoted EMT in the *in vitro* and *in vivo* models of HCC. The above findings suggest the promotive effect of SNHG17 in HCC progression.

LncRNA-miRNA-mRNA molecular signatures are important in various biological processes (Paraskevopoulou and Hatzigeorgiou, 2016; Huang Y.A. et al., 2018; Zhang G. et al., 2018). SNHG17 regulates cell functions by acting as a molecular sponge for miRNAs in breast cancer (Du et al., 2020), tongue squamous cell carcinoma (Liu et al., 2020), and glioma (Li et al., 2020). Using bioinformatic analyses, AGO2-RIP, luciferase reporter assays, and RNA pull-down assays, we demonstrated that SNHG17 sponges miR-3180-3p and inhibits its expression. The role of miR-3180-3p on HCC progression has not been investigated, our results showed that miR-3180-3p overexpression inhibited HCC cell proliferation, migration, and invasion. Subsequently, we found that miR-3180-3p reversed the oncogenic role of SNHG17. Subsequently, we observed that miR-3180-3p targeted and negatively regulated RFX1 functions in HCC cells. Moreover, the expression of RFX1 in HCC tumor tissues correlated with tumor size and Edmonson-Steiner grade. Using CCK-8 and Transwell assays, we showed that the oncogenic role of SNHG17 in HCC was partially exerted via the miR-3180-3p/RFX1 axis.

In conclusion, SNHG17 was upregulated in HCC tumor tissues. Overexpression of SNHG17 promoted HCC cell proliferation, invasion, and migration *in vitro* and *in vivo*. SNHG17 sponged miR-3180-3p, thereby regulating its functions and upregulating RFX1 in the progression of HCC. Taken together, our findings may provide new insights into the molecular mechanisms involved in HCC and use of the SNHG17/miR-3180-3p/RFX1 axis as a promising therapeutic target for HCC.

## Data Availability Statement

The original contributions presented in the study are included in the article/Supplementary Material, further inquiries can be directed to the corresponding author/s.

## Ethics Statement

The studies involving human participants were reviewed and approved by the Hospital Affiliated to Shaanxi University of Chinese Medicine. The patients/participants provided their written informed consent to participate in this study. The animal study was reviewed and approved by The Hospital Affiliated to Shaanxi University of Chinese Medicine.

## Author Contributions

X-XM, JL, and ZC designed the study. TM, XZ, HW, and SY performed the experiments. TM, XZ, YH, YL, HG, and QL participated in the data analysis. HW wrote the manuscript. JL revised the manuscript. All authors approved the final proof.

## Conflict of Interest

The authors declare that the research was conducted in the absence of any commercial or financial relationships that could be construed as a potential conflict of interest.
