# Supplementary material for: Long Non-coding RNA SNHG17 Upregulates RFX1 by Sponging miR-3180-3p and Promotes Cellular Function in Hepatocellular Carcinoma
Source: Front Genet. 2021 Jan 15;11:607636. doi: 10.3389/fgene.2020.607636 (PMC7844393; doi:10.3389/fgene.2020.607636)
Supplement: Supplementary file 1 [file Data_Sheet_1.PDF]

This file includes:

- Supplementary Figures S1

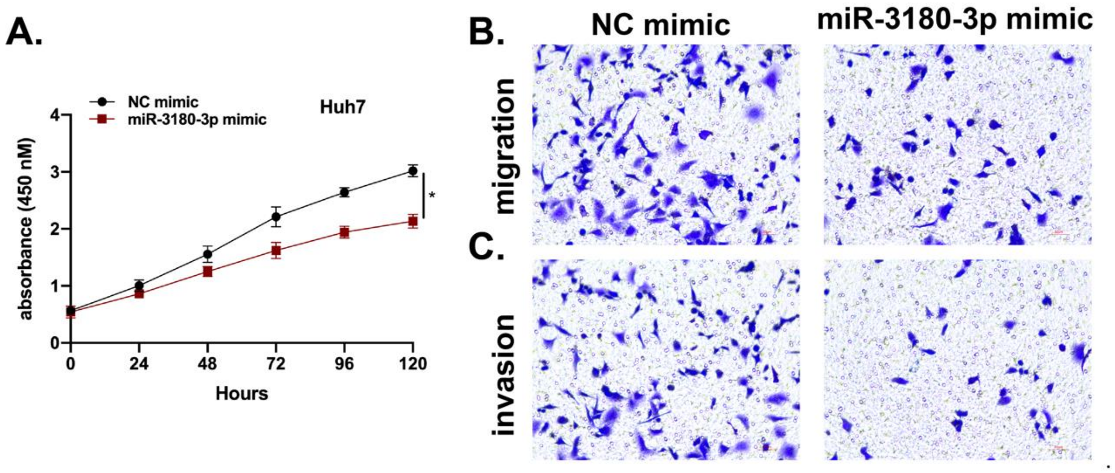

Supplementary Figures S1. The effects of miR-3180-3p on HCC cellular progression. Huh7 cells were stably transfected with miR-3180-3p mimics and its normal control mimics. A: CCK-8 assay was conducted to evaluate the proliferation ability of miR-3180-3p overexpressed Huh7 cells. B: Cell migration level was assessed using transwell migration assay. C: Cell invasion ability was measured by transwell invasion assay. Experiments were repeated three times. *$P < 0.05$.
